# The Preventing Dementia MOOC: Contribution to First Nations’ Health and Well-Being

**DOI:** 10.1093/geroni/igaa057.036

**Published:** 2020-12-16

**Authors:** Lynette Goldberg, Dianne Baldock

**Affiliations:** 1 University of Tasmania, Hobart, Tasmania, Australia; 2 CHAC, Smithton, Tasmania, Australia, United States

## Abstract

Dementia is a global public health issue. First Nations people are at increased risk due to complex intergenerational factors grounded in inequalities in health services and economic and educational opportunities. While there is yet no drug-related cure for this progressive and terminal neurological condition, evidence confirms that increased understanding of dementia and modification of lifestyle factors can reduce risk. The primary potentially modifiable risk factors are not completing secondary school, midlife hypertension, obesity, type II diabetes, depression, physical inactivity, smoking, hearing loss acquired after the age of 55 years, and social isolation. Inherent in these factors is stress, affecting mental health. Addressing these factors globally could prevent or delay over 40 million cases of dementia. The free Preventing Dementia Massive Open Online Course (PD MOOC) is a globally recognized 4-week course that aims to build self-efficacy in knowledge and management of modifiable risk factors. The course has reached over 68,000 people world-wide and is rated highly; however, its contribution to First Nations communities has not yet been investigated. We describe the content of the PD MOOC, report on its impact in a cohort of older Aboriginal people (≥ 50 years of age) in Circular Head, Tasmania, Australia six months after course completion, and emphasize the importance of including traditional approaches to healing. We describe a protocol in which cultural determinants of health can be infused into the PD MOOC and evaluated to promote health and well-being globally for older First Nations people.

